# POEM for Zenker’s diverticulum (Z-POEM): Technical advances, challenges and complications – a narrative review

**DOI:** 10.1016/j.clinsp.2026.100897

**Published:** 2026-03-03

**Authors:** Ygor Rocha Fernandes, Mateus Pereira Funari, Christiano Makoto Sakai, Fauze Maluf-Filho

**Affiliations:** a Department of Gastroenterology, Endoscopy Unit, Hospital das Clínicas, Faculdade de Medicina, Universidade de São Paulo (HCFMUSP), São Paulo, SP, Brazil; b Scholar for National Council for Scientific and Technological Development – CNPq, Brazil

**Keywords:** Zenker’s diverticulum, Peroral Endoscopic Myotomy, Z-POEM, Third-space endoscopy, Flexible Endoscopic Septotomy

## Abstract

**Background/Aims:**

Zenker's Diverticulum (ZD) is a pharyngoesophageal pulsion diverticulum traditionally managed with open surgical or endoscopic (rigid and flexible) techniques. Zenker’s Peroral Endoscopic Myotomy (Z-POEM) has recently emerged as a minimally invasive alternative demonstrating promising efficacy and safety. This narrative review aims to synthesize current evidence regarding the technical evolution of Z-POEM, its indications, procedural nuances, long-term outcomes, cost-effectiveness, and future directions.

**Methods:**

A structured narrative review was conducted in accordance with SANRA guidelines to assess the technical modifications, clinical outcomes, and complications of Zenker’s Per-Oral Endoscopic Myotomy (Z-POEM). Relevant literature published between January 2016 and September 2025 was systematically searched across PubMed, Scopus, and EMBASE, supplemented by manual citation tracing. Eligible studies included clinical trials, cohorts, observational reports, and technical notes focusing on Z-POEM or its variants. Data extraction and synthesis were performed independently by two reviewers, with findings organized into thematic domains comprising procedural techniques, efficacy, safety, adverse events, recurrence, and follow-up.

**Results:**

Proper patient selection is critical for technical success. Factors such as diverticulum size, symptom severity, and anatomical features guide the optimal technique. Long-term studies report sustained clinical success exceeding 85% over 30‒40 months. Repeat Z-POEM procedures have shown high efficacy in managing recurrence. Comparative data indicate Z-POEM achieves superior clinical outcomes compared with flexible endoscopic septotomy, with comparable safety and procedure times.

**Conclusions:**

Z-POEM demonstrates high safety and efficacy, though long-term durability may favor Z-POEM in larger or fibrotic diverticula. Further research should focus on cost-effectiveness, technique standardization, and long-term comparative outcomes to refine patient selection criteria and consolidate Z-POEM as a first-line therapy for ZD.

## Highlights

• Z-POEM achieves >90 % clinical success with durable symptom relief.
• Technical variations (NiZ-POEM, tunnel-free) broaden clinical applicability.
• Safety profile is favorable, with low rates of bleeding or perforation.
• Z-POEM compares favorably to flexible septotomy in efficacy and safety.
• Cost-effectiveness analyses support Z-POEM in specialized centers.

## Introduction

Zenker’s Diverticulum (ZD) is a pharyngoesophageal pulsion diverticulum that is characterized by posterior herniation of the mucosa and submucosa through the Killian’s triangle, the anatomical weak point between the cricopharyngeus and thyropharyngeus muscles.^1^ It typically presents in elderly patients, with the majority of cases occurring in patients over 70-years old.^2^ The pathophysiology of ZD involves altered compliance of the cricopharyngeus muscle, likely caused by fibrotic changes and increased intrabolus pressure.^3^^,^^4^ GERD is suspected to contribute to these fibrotic changes.^5^ The clinical burden of ZD is significant due to symptoms such as dysphagia, regurgitation of undigested food, chronic cough, halitosis, and, in severe cases, weight loss and aspiration pneumonia. These symptoms not only affect quality of life but also pose serious health risks, particularly in ageing populations.^2^^,^^3^^,^^6^

Traditionally, ZD has been managed by open surgical techniques, including cricopharyngeal myotomy combined with diverticulectomy, diverticulopexy, or inversion.^7^^,^^8^ Although effective, these procedures involve cervical incision, general anesthesia, and age-related perioperative morbidity. Endoscopic alternatives emerged in response to these limitations, beginning with rigid endoscopic approaches and eventually incorporating flexible endoscopic instruments.^9^ Harmonic scalpel diverticulotomy, CO_2_-laser myotomy, endoscopic stapler-assisted diverticulostomy, and Flexible Endoscopic Septotomy (FES) have demonstrated clinical success rates ranging from 85 % to 95 %.^9^^,^^10^

Embracing minimally invasive and third-space endoscopy principles, Z-POEM enables precise division of the cricopharyngeus muscle under direct visualization, offering improved control and potentially a lower recurrence rate.^11^^,^^12^ However, to optimize technical challenges due to anatomical constraints, limited working space, and the risk of mucosal injury, various technical modifications were elaborated, such as precut myotomy, tunnel-free Z-POEM, and non-injection variants.^13^^,^^14^

While these innovations broaden the applicability of Z-POEM, they also introduce variability in technique and outcomes.^15^^,^^16^ Currently, no standardized protocol exists for modified Z-POEM, and evidence is largely based on small case series. As adoption grows, a critical appraisal of these evolving techniques is essential. This review aims to synthesize current evidence on technical modifications to Z-POEM, examining their procedural nuances, safety profiles, clinical outcomes, and implications for future standardization and training.

## Methods

A structured narrative review was conducted according to the SANRA (Scale for the Assessment of Narrative Review Articles) guidelines.^17^ to evaluate technical modifications, outcomes, and complications associated with Zenker’s Per-Oral Endoscopic Myotomy (Z-POEM), covering studies published between January 2016 and September 2025. A detailed literature search was performed across PubMed, Scopus, and EMBASE, using combinations of MeSH terms and keywords including “Zenker’s diverticulum”, “Z-POEM”, “third-space endoscopy”, “precut myotomy”, “non-tunnel Z-POEM”, and “POED”, along with outcome-related terms such as “efficacy”, “safety”, “adverse events”, and “complications”. Detailed search strings are provided in the Supplementary File 1 for each searched database. A manual literature search was also conducted via forward citation and backward citation tracing. Searches were tailored to each database’s syntax, and reference lists of key articles were manually screened to identify additional studies.

**Fig. 1:**
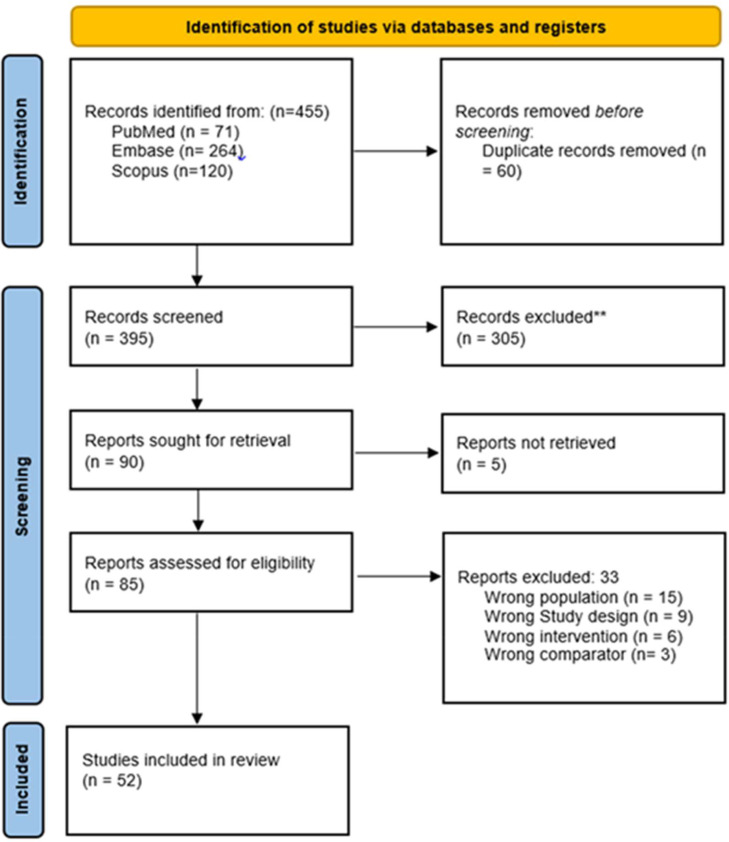
PRISMA Flowchart of study selection process.

Primary and full-text screening was conducted in Rayyan,^18^ and the relevant studies were included in the narrative review. The authors included all the clinical trials, cohort studies, observational reports, or technical notes that focused on Z-POEM or its variants and reported technical details, outcomes, or adverse events. Exclusion criteria included non-endoscopic approaches, other endoscopic techniques, non-English publications, conference abstracts, or editorials lacking full-text data. The PRISMA flow chart for the study selection process is shown in Fig. 1.

Data extraction was guided by a structured reading of eligible studies, with two reviewers independently summarizing relevant content. Discrepancies were resolved by consensus with the senior author. Extracted information was thematically grouped into the following domains that emerged from the literature: technical strategies and procedural modifications, clinical and procedural outcomes, safety and complications, mean diverticula size in mm, Adverse Events (AEs), recurrence, best applications, limitations, and follow-up duration.

### Technical evolution, patient selection, and procedural techniques in Z-POEM

The Z-POEM technique continues to evolve in response to ongoing efforts to improve safety, efficacy, and accessibility for a broader range of patients with ZD.^19^ At its core, Z-POEM builds on the same ideas as POEM, which was originally designed for achalasia.^11^ The procedure starts with a mucosal incision followed by the creation of a submucosal tunnel. This step facilitates exposure of the cricopharyngeal muscle, which is subsequently divided under direct endoscopic visualization. The technique allows for precise myotomy while preserving the mucosal layer on both diverticular and oesophageal sides.^11^^,^^12^

Appropriate patient selection is critical for the success of Z-POEM and its variants.^20^^,^^21^ The procedure is primarily indicated for patients with symptomatic ZD.^2^^,^^3^ Accurate assessment of the diverticulum’s size and orientation is essential for procedural planning.^5^ Barium swallow remains the diagnostic gold standard, as it delineates the size, location, and morphology of the pouch^2^^,^^3^ while endoscopic evaluation provides additional information on mucosal integrity and malignancy exclusion.^9^

Anatomical variations such as anterior diverticula, tortuous pouches, or fibrotic septa from prior interventions increase the difficulty of dissection and tunneling.^2^^,^^9^ In these patients, experienced endoscopists may prefer modified approaches like precut Z-POEM to minimize procedural risk.^13^^,^^22^ Preprocedural CT or MRI may be considered in patients with previous surgery or radiation therapy to assess tissue planes and fibrosis.^4^ Clinical outcomes are closely tied to patient selection. Large diverticula typically require standard tunneling for full myotomy,^23^ while smaller pouches often respond well to simplified techniques such as precut or POED.^12^^,^^14^^,^^21^^,^^24^ Patients with significant comorbidities, prior surgeries, or radiation exposure may benefit most from minimally invasive and modified approaches.^25^ Pre-procedural imaging and multidisciplinary evaluation are critical for determining the most appropriate technique (Table 1) and minimizing procedural risk.^9^^,^^26^^,^^27^

**Table 1:** Comparison of Z-POEM technical variations.

Aspect	Standard Z-POEM	Single-Tunnel Z-POEM	Tunnel-Free Z-POEM	Non-Injection, Non-Tunnel Z-POEM (NiZ-POEM)
Procedure Steps	Dual tunneling, septotomy, clip closure^30^^,^^35^	Unilateral tunnel + esophageal cushion^30^^,^^34^	Bilateral cushions, direct septotomy^19^^,^^45^	Superficial incision, scissor myotomy^31^
Sample Size (n)	22‒89 (multiples series)	8	20	5
Technical Success	87.5 %–100 %^30^^,^^35^	100 %^30^^,^^34^	100 %^19^	100 %^31^
Clinical Success	92 %–100 %^35^	100 %^30^^,^^34^	100 %^19^	100 %^31^
Adverse Events	Perforation (< 5 %), minor bleeding^23^^,^^35^	Mucosal injury (0–5 %)^34^	Aspiration pneumonia (5 %)^19^	None reported^31^
Procedure Time	45–60 min^19^^,^^35^	29–40 min^19^^,^^30^	18–43 min^19^	20–30 min^31^
Key Advantages	Gold standard, deep myotomy^30^^,^^35^	Reduced complexity^30^^,^^34^	Speed, no tunneling^19^	Simplicity, no injection/tunneling^31^
Limitations	Longer duration, technical complexity^23^^,^^35^	Risk of cushion collapse^34^	Risk of cushion collapse, limited long-term data^19^	Small diverticula only, early-stage data^31^

The size of the Zenker’s diverticulum (ZD) may also influence the appropriate endoscopic approach.^20^^,^^21^^,^^28^, ^29^, ^30^, ^31^, ^32^, ^33^ In small diverticula (<2 cm), standard tunneling techniques may be technically challenging due to limited working space. In such cases, simplified approaches such as precut Z-POEM or non-injection non-tunnel Z-POEM (NiZ-POEM) are generally preferred, as they facilitate easier access and minimize technical complexity.^22^^,^^34^ For moderate-sized ZD (2–4 cm), multiple techniques are feasible. Operator experience and anatomical considerations typically guide the choice between Peroral Endoscopic Diverticulotomy (POED) and standard Z-POEM.^29^ In contrast, large diverticula (> 4 cm) often require a full-length myotomy and secure mucosal closure, for which standard Z-POEM is traditionally considered the most appropriate option.^23^ However, emerging evidence suggests that modified techniques such as Tunnel-Free Z-POEM and POED may also be effective in select cases of large diverticula, particularly when performed by experienced endoscopists and tailored to individual anatomical factors.^19^^,^^29^

### Standard Z-POEM (dual-tunnel technique)

The standard Z-POEM technique, first described by Li et al. in 2016, follows a sequence of well-defined steps aimed at achieving complete septotomy under mucosal protection.^35^ The procedure is performed under general anesthesia with endotracheal intubation, using CO_2_ low-flow insufflation to reduce the risk of mediastinal emphysema or pneumoperitoneum.^35^, ^36^, ^37^ A submucosal injection of saline mixed with dye (typically indigo carmine or methylene blue) is administered 1–3 cm proximal to the septum, followed by a 1.5–2 cm longitudinal mucosal incision to access the submucosal layer. Tunneling is then performed on both esophageal and diverticular sides of the septum, exposing the cricopharyngeal muscle. This step is facilitated by the hybrid knives, which combine injection, dissection, and coagulation capabilities.^35^^,^^36^

**Fig. 2:**
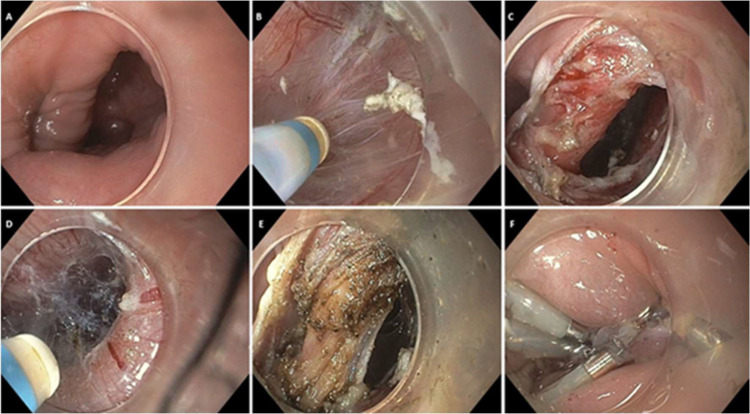
Steps of modified Z-POEM with direct septal incision. (a) Endoscopic visualization of the Zenker's diverticulum septum, with the esophagus (narrower lumen) on the left and the diverticulum (wider lumen) on the right. (b) Submucosal tunneling on the diverticular side. (c) Exposure of the cricopharyngeal muscle forming the septum. (d) Submucosal tunneling on the esophageal side. (e) Myotomy. (f) Complete closure of the mucosal tunnel opening using multiple endoscopic clips.

Once the muscle is exposed, complete myotomy is carried out under direct endoscopic visualization, typically extending to the base of the diverticulum and occasionally including partial dissection of esophageal longitudinal fibers to ensure durability.^2^^,^^35^^,^^36^ The mucosotomy is then closed using Through-The-Scope (TTS) clips. In larger or high-tension defects, Over-the-Scope Clips (OTSC) or endoscopic suturing devices may be employed.^30^ Proper closure is confirmed endoscopically and, if indicated, by post-procedure esophagram.^35^^,^^36^ This standard dual-tunnel technique has demonstrated high technical and clinical success rates exceeding 90 %, particularly in expert centers.^20^^,^^21^^,^^28^ However, it might be time-consuming and technically demanding, especially in patients with small pouches, fibrosis from prior interventions, or altered anatomy,^13^^,^^29^ which has led to the development of simplified variations, as shown in Fig. 2, described in detail in the following section.

### Evolution of Z-POEM techniques

1) Single-tunnel Z-POEM was developed to simplify the procedure. This modification involves creating a mucosotomy directly over the septum and forming a submucosal tunnel on only the diverticular side. To protect the esophageal side, a submucosal cushion is maintained through repeated saline injections. By eliminating the need for bilateral tunneling, this approach reduces procedural time to a median of 33-minutes.^30^ However, there's a risk of esophageal mucosal injury if the cushion is not adequately maintained. Single-tunnel Z-POEM is particularly beneficial in situations with limited working space or fibrosis in one of the diverticular sides
2) Tunnel-free Z-POEM represents a further simplification of the original technique by completely eliminating submucosal dissection. In this technique, bilateral submucosal cushions are created via injections, enabling direct septotomy under endoscopic visualization, with a 2 cm extension into the esophageal muscle.^19^^,^^31^^,^^32^ This approach reduces procedural time to a mean of 30-minutes. While long-term data are still limited, early results suggest that tunnel-free Z-POEM offers comparable efficacy to standard Z-POEM with fewer technical demands.^19^
3) Non-Injection, Non-Tunnel Z-POEM (NiZ-POEM) is an ultra-simplified technique that bypasses both submucosal injection and tunnelling. This method involves using a scissor-type knife to make a superficial mucosal incision, followed by selective grasping and division of the cricopharyngeal muscle without submucosal injection. NiZ-POEM achieves procedural times of 20–30 min with significant technical and clinical success. Currently, no adverse events have been reported with NiZ-POEM. However, its applicability is limited to small diverticula, cases with significant fibrosis, and long-term durability is still under investigation.^31^
4) Precut Z-POEM, introduced by Lajin and colleagues, simplifies tunnel entry by initiating partial myotomy of the septum before completing submucosal tunnelling. This approach improves visualization and facilitates endoscope advancement into the submucosal space, especially in narrow diverticula. Additionally, it may reduce tension at the tunnel entry, aiding mucosal closure and decreasing procedure time. Initial case reports suggest that precut Z-POEM is equally effective as the standard technique, particularly for diverticula measuring <3 cm. Importantly, the technique maintains the core principle of complete septotomy, critical for durable symptom relief.^13^
5) Peroral Endoscopic Diverticulotomy **(**POED) is a hybrid method incorporating principles of Flexible Endoscopic Septotomy (FES) and Z-POEM. It involves shallow tunneling or direct exposure of the septum, followed by complete muscular division. This approach balances procedural simplicity with the benefits of endoscopic precision. Budnicka et al. demonstrated favorable outcomes with POED, particularly in diverticula between 2 and 4 cm. POED might decrease procedural time and facilitate mucosal closure.^20^^,^^29^

## Clinical outcomes and efficacy

Short-term outcomes are defined as clinical results observed within the first 6-months after the procedure, whereas long-term outcomes refer to follow-up data extending beyond 12-months. This section explores the efficacy of Z-POEM in achieving symptom relief, the durability of these results, and the potential factors influencing recurrence.

### Symptom resolution and short-term efficacy

Across the literature, Z-POEM has shown high short-term efficacy, with symptom resolution rates ranging from 90 % to 97 %.^20^^,^^21^^,^^28^^,^^29^ A multicenter study by Elkholy et al. reported that 93.3 % of patients achieved complete resolution of dysphagia within six months.^21^ Similarly, Budnicka et al. noted short-term clinical success in 95 % of treatment-naïve patients, irrespective of the technique that was used.^20^ Precut Z-POEM, as detailed in Lajin’s case report of an 85-year-old woman with a 2 cm diverticulum, demonstrated no adverse events and complete resolution of dysphagia at follow-up.^13^ POED and NIZ-POEM approaches have also yielded comparable symptom relief rates in short-term follow-up, though comparative long-term data remain limited.^29^^,^^31^ NIZ-POEM achieves 100 % technical and clinical success in initial cases with procedural times of 20–30 min and no reported adverse events, though long-term durability is under investigation.^31^

### Long-term outcomes and recurrence

In a retrospective study by Steinway et al., involving 89 patients, a technical success rate of 97.8 % and a clinical success rate of 94 % were reported over an average follow-up of 37-months, with sustained improvements in dysphagia, regurgitation, and respiratory symptoms.^28^ Recurrence rates after Z-POEM typically range from 3 % to 10 % over 12- to 24-months.^20^^,^^21^^,^^24^^,^^28^ Steinway et al. further noted that durable symptom control was maintained in over 85 % of patients at 18-months.^28^ The causes of recurrence include incomplete myotomy, suboptimal tunnel closure, and muscle regrowth.^20^^,^^28^^,^^35^ It is doubtful if modified approaches will enhance myotomy precision. However, the simplified closure by these options might mitigate emphysema, leaks, and mediastinitis, helping to achieve better outcomes.^13^^,^^29^

### Recurrence rates and predictors

A systematic review and meta-analysis reported a recurrence rate of 6.7 % during follow-up. Importantly, recurrences were not significantly associated with incision site or diverticulum size.^10^^,^^27^ Although the mean follow-up period in that study was approximately 13.5-months, these findings support the short- to mid-term durability of Z-POEM’s therapeutic effect, with a low incidence of symptom recurrence.^28^ Modified approaches like Single-Tunnel and Tunnel-Free Z-POEM show comparable early efficacy with 100 % technical success, improvement in dysphagia score from 3 to 0.05 with a significant p-value <0.0001, though long-term data are still lacking.^19^

### Management of recurrent cases

In patients experiencing symptom recurrence, repeat Z-POEM has proven to be both feasible and effective.^38^ One study focusing on patients with prior failed interventions reported a technical success rate of 93.8 % and a clinical success rate of 96.7 % for repeat procedures. Adverse events were minimal and manageable, indicating that Z-POEM remains a viable option even after previous treatment failures.^28^

### Safety profile and complications

Z-POEM is generally considered safe, with a low incidence of serious complications. Commonly reported adverse events include mucosal injuries (5 %–10 %), minor bleeding (3 %–8 %), and pneumomediastinum (1 %–3 %).^23^^,^^39^ Rare but severe events, such as mediastinitis and perforation, occur in fewer than 2 % of cases.^26^ In Lajin’s report, no complications were observed, underscoring the potential safety of modified techniques.^13^ Budnicka et al. noted a 6.8 % complication rate, with most adverse events being minor and conservatively managed.^20^ The technique used can influence complication rates.^40^ Standard Z-POEM (Dual-Tunnel Technique), with technical success rates of 87.5 %–100 % and clinical success of 92 %–100 %, is associated with longer procedural times (45–60 min) but maintains a favorable safety profile in experienced hands, with perforation rates <5 % and minor bleeding in approximately 7 % of cases.^35^ Precut Z-POEM offers a faster, more targeted approach with reduced mucosal trauma.^13^^,^^33^

The most common intraoperative complication is mucosal injury,^21^ which, if recognized early, can usually be closed with TTS clips or endoscopic sutures.^13^^,^^14^ Most mucosal injuries are small and respond well to conservative management.^20^ Pneumomediastinum and delayed perforation, though rare, may result from incomplete closure or unnoticed breaches.^10^^,^^26^ Pneumomediastinum is typically self-limiting,^26^ but delayed perforations can lead to sepsis and require urgent imaging, intravenous antibiotics, and occasionally surgical intervention.^35^ Preventive strategies include using CO_2_ instead of air for insufflation,^37^ administering prophylactic antibiotics,^2^ applying transparent caps for visualization,^32^ and ensuring meticulous mucosal closure.^14^

Single-Tunnel Z-POEM (refer to Table 2) reduces procedural time to 33 min (median) and achieves 100 % technical/clinical success in smaller studies, though esophageal mucosal injury may occur (0–5 %) if the cushion is inadequately maintained.^30^ Tunnel-Free Z-POEM (R-POES/TF Z-POEM) further shortens the) time to 30-minutes (mean) with 100 % success rates, though one study reported aspiration pneumonia (5 %) due to clip obstruction.^19^^,^^31^^,^^32^ NIZ-POEM, which avoids tunneling, has no adverse events reported in initial studies, though it requires a very specific device (scissors-type knife).^31^ Closure methods significantly influence safety outcomes. Inadequate mucosal closure can lead to leaks, emphysema, or mediastinitis.^26^ Effective closure using standard TTS clips or over-the-scope devices is essential, especially in high-tension or fibrotic tissues.^14^^,^^41^

**Table 2:** Comparative outcomes and characteristics of Z-POEM variants.

Technique	Reference	Sample Size	Technical Success	Clinical Success	Procedure time	Mean Diverticulum size (mm)	Adverse Events (AEs)	Recurrence	Follow-up Period	Best applications	Limitations
Standard Z-POEM	Steinway et al., 202,3^12^	89	97.8 %	94 %	61 min	34 ± 13	9 % AEs (3 mild, 5 moderate)	6.7 %	37 months	Large ZD (up to 7 cm); Treatment- naïve patients	Technically demanding in small pouches, fibrotic tissue, or distorted anatomy^9^^,^^15^
Standard Z-POEM	Budnicka et al., 202,1^13^	22	100 %	90.9 %	48.8 ± 19.4 min	30	13.6 % (2 mild emphysema, 1 moderate emphysema with edema)	0 %	8.74 months	Intermediate-sized ZD (2–4 cm)	Limited applicability in recurrent cases^13^
Standard Z-POEM	Elkholy et al., 202,1^14^	24	100 %	95.8 %	43.8 ± 19.2 min	40	20.8 % (subcutaneous emphysema)	4.2 %	10 months	High-risk surgical candidates with symptomatic ZD	Requires expertise in submucosal tunneling ^14^
Precut Z-POEM	Lajin et al., 20,21^8^	1	100 %	100 %	Not reported	20	None	0 %	1 week	Narrow diverticula; cases with limited working space	Case report only; lacks generalizability^8^
Tunnel-Free Z- POEM	Mavrogenis et al., 202,4^10^	20	100 %	100 %	Mean 30 min	30 ± 11.2	5 % (aspiration pneumonia due to esophageal clip)	0 %	18.5 months	Fibrotic or recurrent ZD; avoids submucosal tunneling	Risk of mucosal injury; limited long-term data^10^
Open Z-POEM	Fayyaz et al., 202,5^38^	53	100 %	100 %	19 ± 6 min	26 ± 14	3.8 % (1 moderate ulcer, 1 severe leak)	3.8 %	5.4 months	Recurrent ZD; avoiding mucosal closure	Early leak risk; lacks randomized comparison^38^

### Comparative evaluation: Z-POEM vs. FES vs. transcervical surgery

Comparative studies evaluating Z-POEM, FES, and transcervical surgery reveal nuanced differences in safety, efficacy, and procedural logistics. Z-POEM offers enhanced mucosal protection and controlled septotomy, particularly in larger or fibrotic diverticula, with technical success rates exceeding 90 % and clinical success rates of 93 %–95 % over 6–12 months.^42^^,^^43^ FES, while faster (mean time 10–20 min), may be limited by poor visualization and higher recurrence in diverticula > 3 cm.^44^ Transcervical surgery remains effective but carries higher morbidity, longer recovery, and increased risk of complications such as mediastinitis and vocal cord injury. Steinway et al. reported sustained symptom relief in 85 % of Z-POEM patients at 37-months, while recurrence rates for FES ranged from 10 %–15 % in comparable cohorts.^28^ These findings suggest that while FES may offer procedural speed, Z-POEM provides superior anatomical reach and long-term durability, especially in complex cases.

The emergence of Z-POEM modifications, such as Precut, Tunnel-Free, and NiZ-POEM reflects a growing effort to overcome anatomical and procedural challenges posed by Zenker’s Diverticulum (ZD). However, the quality of evidence supporting these innovations remains variable. Most published data derive from small retrospective series or case reports, limiting the strength of conclusions and generalizability.^45^^,^^46^ For instance, while NiZ-POEM offers procedural efficiency by eliminating submucosal injection, its long-term durability remains uncertain, and the technique may compromise the “protected-space” principle central to POEM’s safety profile. Elkholy et al. reported high short-term success rates across Z-POEM variants, but emphasized that incomplete septotomy and suboptimal closure techniques were key contributors to recurrence.^21^ Similarly, Steinway et al. provided ≥ 2-years follow-up data showing that recurrence often correlated with technical shortcuts, such as abbreviated myotomy or superficial dissection.^28^ These findings suggest that the drive for procedural speed must be balanced against long-term outcomes, particularly in high-risk or fibrotic anatomy. The manuscript now incorporates this synthesis to critically appraise whether technique evolution is truly patient-centred or driven by operator convenience.

## Discussion

Despite its promising efficacy, Z-POEM remains technically demanding due to the anatomical challenges posed by ZD. The septum features and variable pouch orientation make submucosal tunnelling technically demanding, particularly in patients with prior procedures or fibrosis.^14^^,^^32^ Limited space, unstable position, poor dissection planes, and reduced tissue pliability increase technical difficulty and the risk of mucosal injury during dissection.^10^^,^^32^ Successful tunnel creation requires frequent saline injections, high-precision instruments like the hybrid knives, and continuous endoscopic visualization.^10^^,^^32^^,^^45^

The cited technical challenges and technological device improvement, along with the purpose for better outcomes, boosted the modified techniques, each one offering specific advantages. Therefore, the application of Z-POEM technique variations should consider the specific case instead of rigid algorithms. For instance, single tunnel Z-POEM may mitigate difficulties in dissecting one of the septum sides, but requires meticulous cushion maintenance to avoid mucosal injury.^30^^,^^38^ NiZ-POEM and Precut Z-POEM are favoured in small diverticula or narrow working spaces due to their reduced procedural complexity and shorter duration.^13^^,^^31^ In moderate-sized pouches (2–4 cm), both POED and standard Z-POEM are viable options, with POED offering a balance of simplicity and efficacy for operators transitioning from flexible septotomy.^20^^,^^29^ Large diverticula (> 4 cm) may benefit from standard Z-POEM, as it allows comprehensive septotomy with reliable mucosal closure in more spacious anatomy.^23^^,^^28^ In patients with prior interventions or significant fibrosis, Tunnel-Free or Precut Z-POEM may minimize dissection challenges by simplifying entry and closure.^13^^,^^19^ Some modified techniques may facilitate mucosotomy closure, decreasing adverse event rates and providing better long-term outcomes. Elderly or high-risk patients often tolerate better simplified, minimally invasive approaches such as NiZ-POEM or Single-Tunnel Z-POEM due to shorter anesthesia times and reduced tissue trauma.^30^^,^^31^ In cases of recurrence or failed prior Z-POEM, repeating standard Z-POEM remains effective, enabling full resection of residual muscle fibers.^28^^,^^38^

Importantly, recurrence following Z-POEM may be influenced by several technical factors inherent to these modifications. Shorter myotomy lengths, as seen in NiZ-POEM or Precut Z-POEM, may leave residual muscle fibers untreated, increasing the likelihood of symptom persistence or relapse.^47^ Inadequate depth of dissection, particularly when septotomy is superficial or incomplete, can also contribute to recurrence.^48^ Closure technique plays a pivotal role; poor mucosal apposition or premature clip detachment may lead to inadequate healing and diverticular reformation.^48^^,^^49^ Furthermore, in fibrotic or previously treated anatomy, limited visualization and reduced tissue pliability may compromise the completeness of myotomy.^19^ A synthesis of available data suggests that recurrence is multifactorial, with technical precision, anatomical complexity, and operator experience all contributing. Future studies should stratify recurrence rates by technique, diverticulum size, and procedural variables to better inform technique selection and optimize long-term outcomes.

This narrative review is subject to several limitations inherent to the available data. First, most of the included studies are retrospective case series with small sample sizes, lacking control groups or randomization, which limits the overall quality and generalizability of the evidence. Second, there is substantial heterogeneity in procedural descriptions across institutions, particularly regarding the extent of myotomy, tunneling strategies, and methods of mucosal closure. This variability complicates direct comparisons and emphasizes the need for standardized protocols, structured training, and comparative studies. Although formal cost-effectiveness analyses are lacking, some authors speculate that simplified Z-POEM techniques, such as NiZ-POEM and Tunnel-Free Z-POEM may reduce anesthesia time and procedural complexity, potentially lowering resource utilization.^49^ However, these assumptions remain speculative and require validation through prospective economic studies.

Future prospective multicenter studies should address these gaps by incorporating standardized outcome metrics, longer follow-up, cost analyses, and head-to-head comparisons with established surgical and flexible endoscopic techniques. Parallel learning curves and proficiency steps also require deeper investigation. Additionally, further evaluation is needed to refine patient selection criteria. Identifying predictors of favorable outcomes, including diverticulum size, anatomical variations, and comorbidity profiles, will enable a more tailored and risk-adapted therapeutic approach. Meanwhile, less robust data from case series and midterm follow-up studies will be interpreted in light of expert experience to inform clinical practice and improve patient outcomes. Preliminary results from small series evaluating Tunnel-Free POEM and NiZ-POEM suggest acceptable short-term safety and symptom relief, with follow-up durations ranging from 3- to 12-months. However, these findings are limited by sample size and lack of long-term durability data. What seems clear from preliminary results is a good safety and efficacy profile, once a satisfactory myotomy is usually achieved. Initial studies of NiZ-POEM report 100 % technical and clinical success in small diverticula, with procedural times of 20 to 30 min and no adverse events over a 6-month follow-up. Similarly, Budnicka et al. demonstrated 95 % symptom resolution with POED in diverticula 2–4 cm, with no major complications.^20^ Elkholy et al. reported 93.3 % clinical success with standard Z-POEM at 6-months in a multicenter cohort of 89 patients.^45^

As previously exposed, third-space endoscopy for treating ZD, summarized as conventional and modified Z-POEM, is a relatively new field in therapeutic endoscopy. Many technical variants offer an advantage in specific conditions, which include anatomy, fibrosis, operator experience, and resource availability. Considering the difficulty of previewing the challenges during the procedure, the ideal scenario would be to know and have proficiency regarding the technical possibilities and to use them according to the concrete case one is facing.

## Conclusion

Z-POEM represents a significant advancement in the management of ZD. As a minimally invasive, safe, and effective alternative to traditional surgical approaches, Z-POEM offers high adaptability through technical modifications that allow for individualized treatment strategies. Long-term data affirm its durability, with high clinical success and low recurrence rates. Although formal cost-effectiveness analyses are lacking, preliminary evidence suggests that simplified Z-POEM variants may reduce anesthesia and procedural time, potentially lowering resource utilization. However, further research is warranted to standardize techniques, establish comprehensive cost-effectiveness analyses, and conduct randomized controlled trials. These efforts will help solidify Z-POEM's role as a first-line therapy and guide best practices in ZD management. The development of standardized protocols for technique selection and procedural steps will be essential for optimizing outcomes and training.

## Ethical approval

Not required.

## Informed consent

Not applicable.

## Peer review and provenance statement

Not applicable.

## Availability of data and materials

No datasets were generated or analyzed during the current study.

## Ethical statement

Not applicable.

## Data availability

Data sharing is not applicable to this article as no new datasets were generated or analyzed. All data supporting the findings of this narrative review are available within the article and derive from previously published studies, which are fully cited in the reference list.

## Authors' contributions

Conceptualization: Ygor Rocha Fernandes; Methodology: Ygor Rocha Fernandes, Mateus Pereira Funari; Writing-original draft: Ygor Rocha Fernandes, Mateus Pereira Funari; Writing-review & editing: Ygor Rocha Fernandes, Christiano Makoto Sakai, Fauze Maluf-Filho; Supervision: Fauze Maluf-Filho.

## Funding

None.

## Declaration of competing interest

The authors declare that they have no known competing financial interests or personal relationships that could have influenced the work reported in this paper.

## Appendix Supplementary materials

Image, application 1

## Footnotes

Supplementary material associated with this article can be found, in the online version, at doi:10.1016/j.clinsp.2026.100897.

## References

[1] Law R., Katzka D.A., Baron T.H. (2014). Zenker's Diverticulum. Clin Gastroenterol Hepatol.

[2] Ferreira L.E., Simmons D.T., Baron T.H. (2008). Zenker's diverticula: pathophysiology, clinical presentation, and flexible endoscopic management. Dis Esophagus.

[3] Siddiq M.A., Sood S., Strachan D. (2001). Pharyngeal pouch (Zenker's diverticulum). Postgrad Med J.

[4] Hussain T., Maurer J.T., Lang S., Stuck B.A. (2017). Pathophysiology, diagnosis and treatment of Zenker's diverticulum]. HNO.

[5] Sirasapalli S.K., Senthamizhselvan K., Mohan P. (2023). Rare association of Killian-Jamieson diverticulum and peptic stricture of the esophagus: is it causal or casual?. Euroasian J Hepatogastroenterol.

[6] Martinez Paredes J.F., Al Fakir R., Rutt A.L (2022). Clinical symptoms contributing to Zenker's diverticulum repair: a retrospective review. Cureus.

[7] Kaminski M.F., Budnicka A., Przybysz A. (2024). Pilonis ND. Traditional septotomy or Z-POEM for Zenker's diverticulum. Best Pract Res Clin Gastroenterol.

[8] Epping H., Ziachehabi A., Spaun G., Wewalka F., Maieron A., Schöfl R. (2022). Flexible diverticulotomy for Zenker's diverticulum ‒ a bicentric study. Z Gastroenterol.

[9] Dell'Anna G., Fasulo E., Fanizza J., Barà R., Vespa E., Barchi A. (2024). The Endoscopic Management of Zenker's Diverticulum: a comprehensive review. Diagnostics (Basel).

[10] Costamagna G., Iacopini F., Bizzotto A., Familiari P., Tringali A., Perri V. (2016). Prognostic variables for the clinical success of flexible endoscopic septotomy of Zenker's diverticulum. Gastrointest Endosc.

[11] Singh S., Chandan S., Bapaye J., Brar H.S., Mohammed A., Kassab L.L. (2025). Peroral endoscopic myotomy (Z-POEM) versus flexible endoscopic septotomy (FES) for treatment of Zenker's diverticulum: does either make the cut? A systematic review and meta-analysis of outcomes. Ann Gastroenterol.

[12] Benites-Goñi H., Bardalez-Cruz P., Medina-Morales B., Asencios-Cusihuallpa J., Marin-Calderón L. (2024). Peroral endoscopic septotomy for Zenker's diverticulum with additional cut of mucosal flap: step by step. VideoGIE.

[13] Lajin M. (2021). Modified Z-POEM technique to allow easier closure of the tunnel entry. Endosc Int Open.

[14] Klingler M.J., Landreneau J.P., Strong A.T., Barajas-Gamboa J.S., Tat C., Tu C. (2021). Endoscopic mucosal incision and muscle interruption (MIMI) for the treatment of Zenker's diverticulum. Surg Endosc.

[15] El Abiad R., Brindise E., Bejjani M., Ghandour B., Khashab M. (2023). Peroral endoscopic myotomy and septotomy for Zenker diverticulum. Minerva Gastroenterol (Torino).

[16] Repici A., Spadaccini M., Belletrutti P.J., Galtieri P.A., Fugazza A., Anderloni A. (2020). Peroral endoscopic septotomy for short-septum Zenker's diverticulum. Endoscopy.

[17] Baethge C., Goldbeck-Wood S., Mertens S. (2019). SANRA-a scale for the quality assessment of narrative review articles. Res Integr Peer Rev.

[18] Ouzzani M., Hammady H., Fedorowicz Z., Elmagarmid A. (2016). Rayyan ‒ a web and mobile app for systematic reviews. Syst Rev.

[19] Mavrogenis G., Zachou M., Tsevgas I., Markoglou K., Zachariadis D., Spanomanoli A. (2024). Tunnel-free peroral endoscopic myotomy reduces procedural time and maintains efficacy in Zenker's diverticulum. Ann Gastroenterol.

[20] Budnicka A., Januszewicz W., Białek A.B., Spychalski M., Reguła J., Kaminski M.F. (2021). Peroral endoscopic myotomy in the management of Zenker's diverticulum: a retrospective multicenter study. J Clin Med.

[21] Elkholy S., El-Sherbiny M., Delano-Alonso R., Herrera-Esquivel J.J., Valenzuela-Salazar C., Rodriguez-Parra A. (2021). Peroral endoscopic myotomy as treatment for Zenker's diverticulum (Z-POEM): a multi-center international study. Esophagus.

[22] Zhang L.Y., Nieto J., Ngamruengphong S., Repici A., Khashab M.A. (2021). Zenker's diverticulum: advancing beyond the tunnel. VideoGIE.

[23] Elkholy S., Essam K., El-Sherbiny M. (2020). Z-POEM (Per Oral Endoscopic Myotomy) for the management of large Zenker's diverticulum. Acta Gastroenterol Belg.

[24] Podgaetz E., Konda V. (2021). Experience and technique for Zenker's diverticulum per oral endoscopic myotomy: Z-POEM. Thorac Cardiovasc Surg.

[25] Nabi Z., Nageshwar Reddy D. (2022). Impact of modified techniques on outcomes of peroral endoscopic myotomy: a narrative review. Front Med (Lausanne).

[26] Case D.J., Baron T.H. (2010). Flexible endoscopic management of Zenker diverticulum: the Mayo Clinic experience. Mayo Clin Proc.

[27] Murat Buyruk A., Erdoğan Ç. (2024). Efficacy and safety of peroral endoscopic myotomy in the treatment of Zenker's diverticulum: a single-center experience. Turk J Gastroenterol.

[28] Steinway S., Zhang L., Amundson J., Nieto J., Desai P., Jacques J. (2023). Long-term outcomes of Zenker's peroral endoscopic myotomy (Z-POEM) for treatment of Zenker's diverticulum. Endosc Int Open.

[29] Pugliese F., Dioscoridi L., Italia A., Forgione A., Cintolo M., Forti E. (2020). Peroral endoscopic diverticulotomy (POED) for Zenker Diverticulum using submucosal injection to perform a complete myotomy. Surg Laparosc Endosc Percutan Tech.

[30] Mavrogenis G., Maurommatis E., Koumentakis C., Tsevgas I., Zachariadis D., Bazerbachi F. (2023). Single-tunnel Zenker's diverticulum peroral endoscopic myotomy. Endoscopy.

[31] Gorrepati V.S., Yang D., Draganov P.V (2023). Novel non-injection non-tunnel technique for peroral endoscopic myotomy of Zenker's diverticulum. VideoGIE.

[32] Estevinho M.M., Pinho R., Rodrigues J., Correia J., Freitas T. (2023). Tunneling-free peroral endoscopic septotomy for Zenker diverticulum. VideoGIE.

[33] Bizzotto A., Iacopini F., Landi R., Costamagna G. (2013). Zenker's diverticulum: exploring treatment options. Acta Otorhinolaryngol Ital.

[34] Young E., Singh R. (2022). Modified Zenker's peroral endoscopic myotomy: a novel technique to improve access and depth of muscular dissection. VideoGIE.

[35] Li Q.L., Chen W.F., Zhang X.C., Cai M.Y., Zhang Y.Q., Hu J.W. (2016). Submucosal tunneling Endoscopic Septum division: a novel technique for treating Zenker's diverticulum. Gastroenterology.

[36] Brewer Gutierrez O.I., Ichkhanian Y., Spadaccini M., Vosoughi K., Repici A., Khashab M.A (2019). Zenker's diverticulum per-oral endoscopic myotomy techniques: changing paradigms. Gastroenterology.

[37] Takada J., Araki H., Onogi F., Nakanishi T., Kubota M., Ibuka T. (2015). Safety and efficacy of carbon dioxide insufflation during gastric endoscopic submucosal dissection. World J Gastroenterol.

[38] Yang J., Novak S., Ujiki M., Hernández Ó., Desai P., Benias P. (2020). An international study on the use of peroral endoscopic myotomy in the management of Zenker's diverticulum. Gastrointest Endosc.

[39] Zhang H., Huang S., Xia H., Shi L., Zeng X., Jiang J. (2022). The role of peroral endoscopic myotomy for Zenker's diverticulum: a systematic review and meta-analysis. Surg Endosc.

[40] Li Q.L., Zhou P.H. (2015). Perspective on peroral endoscopic myotomy for achalasia: zhongshan experience. Gut Liver.

[41] Che S.Y.W., Joseph S., Kuchta K., Amundson J.R., VanDruff V.N., Ishii S. (2024). Outcomes after per-oral endoscopic myotomy for Zenker's diverticula (Z-POEM) and correlation with impedance planimetry (FLIP). Surg Endosc.

[42] Economopoulos K.P., Rothman D.L.P., Witkowski E., Paranjape C., Krishnan K. (2025). Outcomes of Zenker’s peroral endoscopic myotomy (Z-POEM) for treatment of Zenker’s diverticulum at our tertiary care center: a single-institution retrospective cohort study. Surg Endosc.

[43] Norton B., Siggens K., Papaefthymiou A., Telese A., Duku M., Murino A. (2025). Zenker peroral endoscopic myotomy is safe and effective for the management of pharyngeal pouch: a multicentre retrospective cohort study. Digestive Diseases.

[44] Wilmsen J., Baumbach R., Stüker D., Weingart V., Neser F., Gölder S.K. (2017). New flexible endoscopic controlled stapler technique for the treatment of Zenker's diverticulum: a case series. World J Gastroenterol.

[45] Elkholy S., El-Sherbiny M., Delano-Alonso R., JdJ Herrera-Esquivel, Valenzuela-Salazar C., Rodriguez-Parra A. (2021). Peroral endoscopic myotomy as treatment for Zenker’s diverticulum (Z-POEM): a multi-center international study. Esophagus.

[46] Youssef M., Ching Hui Yee C., Bechara R. (2024). A144 Zenker’s Peroral Endoscopic Myotomy (Z-POEM) outcomes in treatment of Zenker’s Diverticulum: a retrospective case series. J Can Assoc Gastroenterol.

[47] Fugazza A., Cappello A., Capogreco A., Repici A., Testoni P.A., Inoue H., Wallace M.B. (2022). Gastrointestinal and Pancreatico-Biliary Diseases: Advanced Diagnostic and Therapeutic Endoscopy.

[48] Ramamurthy S., Ahuja P., Dahiya D.S., Hayat U., Ahuja N., Bharadwaj H.R. (2025). Management Strategies for Zenker’s diverticulum: a comprehensive review. J Clin Med.

[49] Fujiyoshi Y., Onimaru M., Inoue H. (2021). What are the factors for detecting adverse events in second-look endoscopy after per-oral endoscopic myotomy (POEM)? A reply to “second-look endoscopy after POEM for all, some or none… more you see, the more you find!”. Digestive Endoscopy.

