# Supplementary material for: POEM for Zenker’s diverticulum (Z-POEM): Technical advances, challenges and complications – a narrative review
Source: Clinics (Sao Paulo). 2026 Mar 3;81:100897. doi: 10.1016/j.clinsp.2026.100897 (PMC12969442; doi:10.1016/j.clinsp.2026.100897)
Supplement: Supplementary file 1 [file mmc1.docx]

CLINICS-D-25-01404_Supplementary Material

**Supplementary File 1** **Search strategies for database queries.** Detailed search strings used to identify relevant studies across PubMed, Embase, and Scopus databases. The searches were conducted in **September 2025**, without language restrictions, and were limited to human studies published between **January 2016 and September 2025**. The specific search strings and number of results retrieved from each database are summarized as follows: PubMed (71 records), Embase (264 records), and Scopus (120 records).

| **SEARCH STRINGS** |
| --- |
| **Pubmed** |
| **Date of search**: September 2025 |
| **Result**s: 71 |
| **Search string:** |
| ("Zenker Diverticulum"[Mesh] OR "Zenker Diverticulum" OR "Zenker’s diverticulum" OR "pharyngeal pouch") AND ("Peroral Endoscopic Myotomy"[Mesh] OR "peroral endoscopic myotomy" OR "POEM" OR "Z-POEM" OR "Zenker’s peroral endoscopic myotomy" OR "peroral endoscopic septum division" OR "third-space endoscopy" OR "tunnel-free POEM" OR "non-tunnel POEM" OR "single-tunnel Z-POEM" OR "open Z-POEM" OR "NiZ-POEM") AND ("Treatment Outcome"[Mesh] OR "treatment outcome" OR "clinical success" OR "symptom relief" OR "dysphagia score" OR "recurrence" OR "complications" OR "adverse events" OR "safety" OR "procedure time" OR "hospital stay") |
|  |
| **Embase** |
| **Date of search**: September 2025 |
| **Results**: 264 |
| **Search string:** |
| ('zenker diverticulum'/exp OR 'zenker diverticulum' OR 'diverticle zenker' OR 'diverticulum, hypopharyngeal' OR 'diverticulum, pulsion' OR 'diverticulum, pulsion esophageal' OR 'diverticulum, pulsion oesophageal' OR 'diverticulum, zenker' OR 'esophageal diverticulum, hypopharyngeal' OR 'esophagus diverticulum, pulsion' OR 'esophagus pseudodiverticulum' OR 'hypopharyngeal diverticulum' OR 'hypopharyngeal esophageal diverticulum' OR 'hypopharyngeal oesophageal diverticulum' OR 'hypopharynx diverticulum' OR 'oesophageal diverticulum, hypopharyngeal' OR 'pulsion diverticulum, esophageal' OR 'pulsion diverticulum, oesophageal' OR 'zenker diverticle' OR 'zenker pouch' OR 'zenker`s diverticulum') AND ('peroral endoscopic myotomy'/exp OR 'poem (peroral endoscopic myotomy)' OR 'peroral endoscopic myotomy') |
|  |
| **Scopus** |
| **Date of search**: September 2025 |
| **Results**: 120 |
| **Search string:** |
| (TITLE-ABS-KEY ("zenker diverticulum" OR "zenker's diverticulum" OR "pharyngeal pouch" OR "hypopharyngeal diverticulum")) AND (TITLE-ABS-KEY ("peroral endoscopic myotomy" OR POEM OR "Z-POEM" OR "zenker's peroral endoscopic myotomy" OR "peroral endoscopic septum division" OR "third-space endoscopy" OR "tunnel-free POEM" OR "non-tunnel POEM" OR "precut myotomy")) AND (TITLE-ABS-KEY ("treatment outcome" OR "clinical outcome" OR "therapy efficacy" OR "treatment success" OR "symptom relief" OR "dysphagia score" OR recurrence OR safety OR complications OR "adverse events")) |
